# Antibacterial effects and action modes of asiatic acid

**DOI:** 10.7603/s40681-015-0016-7

**Published:** 2015-08-18

**Authors:** Wen-hu Liu, Te-Chung Liu, Mei-chin Mong

**Affiliations:** 1 School of Nutrition, Chung Shan Medical University, 402 Taichung, Taiwan; 2 Department of Health and Nutrition Biotechnology, Asia University, 413 Taichung, Taiwan

**Keywords:** Asiatic acid, Bacterial contamination, Antibacterial, Membrane damage

## Abstract

In this study, the antibacterial effects and action modes of asiatic acid against the foodborne bacterial pathogens *Escherichia coli* O157:H7, *Salmonella* Typhimurium DT104, *Pseudomonas aeruginosa, Listeria monocytogenes, Staphylococcus aureus, Enterococcus faecalis*, and *Bacillus cereus* were examined. Minimal inhibitory concentrations (MICs) of asiatic acid against these bacteria were in the range of 20-40 μg/mL. *Minimum bactericidal concentrations of asiatic acid* were in the range of 32-52 μg/mL. Asiatic acid at 2X MIC effectively reduced bacterial numbers from 6 log_10_ to < 2 log10 in all test bacteria within 6 h (*P* < 0.05). The antibacterial activity of asiatic acid was not affected by heat treatments from 25 to 100°C. Asiatic acid at 1 or 2X MICs caused 40-56% and 71-89% membrane damage in test bacteria within 2 h, respectively In addition, asiatic acid at 1 or 2X MICs led to 1.5-2.4 ppm and 2.9-4.1 ppm K^+^ release within 2 hr, respectively. Asiatic acid treatments dose-dependently increased bacterial nucleotide leakage (*P* < 0.05). After 3 days of storage at 25°C, the addition of asiatic acid dose-dependently inhibited the growth of test bacteria in ground beef (*P* < 0.05), in which 8 mg asiatic acid treatments led to bacterial levels (log CFU/g) in said ground beef lower than 2. These findings suggest that asiatic acid might be a potent antibacterial agent to prevent food contamination.

## 1. Introduction

*Escherichia coli* O157:H7, *Salmonella* Typhimurium DT104, *Pseudomonas aeruginosa, Listeria monocytogenes, Staphylococcus aureus, Enterococcus faecalis*, and *Bacillus cereus* are seven common foodborne bacterial pathogens [1-4]. These bacteria contaminate many foods including meat, seafood, dairy products, and juice [5-8]. It is well known that contamination from these bacteria reduces the shelf-life of foods, and leads to economic loss for food producers. Most importantly, however, food contamination from these bacteria causes foodborne illness in consumers. The foodborne disease outbreaks due to these bacteria in Taiwan and other countries have been well reported [4, 9]. Therefore, the development and application of a proper agent with antibacterial activity would be helpful in ensuring food safety.

**Fig. 1:**
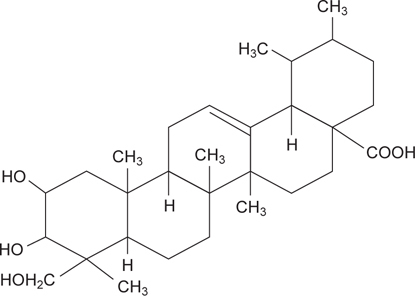
Structure of asiatic acid.

Asiatic acid is a pentacyclic triterpene (Figure 1) that naturally occurs in many vegetables and fruits such as glossy privet fruit (*Ligustrum lucidum* Ait.), basil (*Ocimum basilicum*), and brown mustard (*Brassica juncea*) [10, 11]. It has been reported that this compound exhibits inhibitory effects against *S. aureus, B. cereus, E. coli, B. subtilis*, and *Shigella sonnei*, as determined by agar diffusion methods [12, 13]. Garo et al. indicated that asiatic acid could enhance the susceptibility of *P. aeruginosa* biofilms to tobramycin [14]. The study of Masoko et al. revealed that asiatic acid benefited wound healing *via* its anti-fungal activity [15]. These previous studies imply that asiatic acid is a potent antimicrobial agent; however, it is unknown whether or not asiatic acid could affect the viability of S. Typhimurium DT104, *E. coli* O157:H7, *L. monocytogenes, P. aeruginosa*, and *E. faecalis*. Furthermore, the minimal inhibitory concentrations (MICs) and action modes of asiatic acid against these bacteria remain unclear.

Bacterial cell membrane integrity and permeability play crucial roles for bacterial survival and growth. Thus, any agent with the ability to destroy bacterial cell membrane integrity and/or disrupt membrane permeability may cause bacterial cell damage, and even death. In addition, the rupture of bacterial cytoplasmic membrane promotes the release of intracellular components such as potassium ions and nucleotides, which in turn diminishes bacterial ability to repair and replicate [16, 17]. So far, the assays regarding the variation in bacterial membrane integrity, K^+^ efflux, and nucleotides release have been widely used to examine the antibacterial actions of some select agents [18-20]. Thus, if asiatic acid could cause bacterial membrane damage and/or enhance the release of potassium ions and nucleotides, its antibacterial action could be explained.

The major purpose of this study was to investigate the inhibitory effects of asiatic acid against seven foodborne bacterial pathogens. The influence of this compound upon the membrane damage, potassium ions and nucleotides loss in these bacteria was also evaluated. Ground beef was used as a food model to examine the antibacterial effects of asiatic acid at various doses.

## 2. Materials and methods

### 2.1. Materials

Asiatic acid (98%) was purchased from Sigma-Aldrich Co. (St. Louis, MO, USA). Asiatic acid, based on its hydrophobic characteristic, was first dissolved in dimethyl sulphoxide (DMSO, 20 mg/ml), and then used for other preparations. The final concentration of DMSO in the culture medium was maintained at 0.5% (v/v). The impact of DMSO upon the growth of test bacteria was not significant (data not shown).

### 2.2. Test organisms

Three Gram-negative bacteria, *E. coli* O157:H7, *S*. Typhimurium DT104, *P. aeruginosa*, and 4 Gram-positive bacteria, *L. monocytogenes, S. aureus, E. faecalis* and *B. cereus* were recovered from contaminated chicken, duck, and dairy products, as well as seafood from May, 2012 to August, 2013 by using a surface swab technique. Swab samples were directly streaked onto a Chromocult Coliform agar plate, a Brilliant Green agar plate, a Cetrimide agar plate, a Baird Parker agar plate, a *Listeria* selective agar plate, a brain heart infusion agar plate or *B. cereus* selective agar plate for *E. coli* O157:H7, *S*. Typhimurium DT104, *P. aeruginosa, S. aureus, L. monocytogenes, E. faecalis or B. cereus* enumeration, respectively. These selective agars were purchased from Oxoid Ltd. (Basingstoke, UK). After sample streaking, agar plates were incubated for 24 h at 37°C. One isolated colony from a contaminated food was defined as 1 isolate. In this study, 16 isolates from 16 different contaminated foods for each test bacterial strain were used for experiments.

### 2.3. MIC and minimum bactericidal concentration (MBC) determination

MICs of asiatic acid against test bacteria were determined according to the Clinical and Laboratory Standards Institute guideline [21]. Each bacterial strain culture at 0.1 ml, containing 10^6^ CFU/ ml as determined by plates count, was inoculated into a 9.9 ml Mueller Hinton (MH) broth (Difco, MI, USA) supplemented with asiatic acid at concentrations ranging from 2 to 512 μg/ml in tubes. All tubes were then incubated at 35°C for 24 h in an incubator (Model LE-30D, Yih Der Co., Taipei, Taiwan). MIC was recorded as the lowest concentration of asiatic acid to inhibit visible growth of test bacteria, which was reflected by no variation in turbidity. Turbidity was assayed by an optical density (OD) measurement at 600 nm with a UV spectrophotometer (Model UV-1800, Shimadzu Co., Tokyo, Japan). By sub-culturing from the MIC assay tubes onto MH agar plates and incubating at 35°C for another 24 h, MBC was the lowest concentration of asiatic acid to inhibit visible growth on agar plates. All experiments were performed in triplicate.

**Table 1:** Minimum inhibitory concentration (MIC, μg/ml) and minimum bactericidal concentration (MBC, μg/ml) of asiatic acid against seven bacteria.

Bacteria	MIC	MBC
Gram-negative bacteria		
*E. coli* O157:H7	24 ± 4	36 ± 4
*S*. Typhimurium DT104	32 ± 2	40 ± 4
*P. aeruginosa*	36 ± 4	44 ± 2
Gram-positive bacteria		
*L. monocytogenes*	36 ± 4	48 ± 8
*S. aureus*	28 ± 2	44 ± 4
*E. faecalis*	20 ± 2	32 ± 4
*B. cereus*	40 ± 4	52 ± 2

Data are expressed as mean ± SD (n = 16).

### 2.4. Time-kill study assay

*In vitro* time-kill of asiatic acid at 0.5, 1 and 2X MICs (Table 1) against test bacterial strains was monitored in a 10 ml MH broth at 35°C, after inoculation with culture at 10^6^ CFU/ml. At 0, 3, 6, 9 and 12 h, bacterial suspensions at 100 μl were cultured on MH plates for determination of CFU/ml. The plates were incubated at 35°C for 24 h, and the colonies were counted. The detection limit was 20 CFU/ml.

### 2.5. Heat treatment

A 10 ml beaker with a solution of asiatic acid (20 mg/ml) was sealed with parafilm and placed in a water bath incubator (Model BH-230D, Yih Der Co., Taipei, Taiwan). The temperature of the beakers was maintained at 25, 50, 75 or 100°C for 60 min in this incubator. After cooling down to room temperature, the inhibitory zone of these solutions against the test bacteria was determined.

### 2.6. Inhibitory zone measurement

The inhibitory zone was determined and compared by disc diffusion method. A sterile blank disc (6 mm diameter, Difco, MI, USA) was soaked in asiatic acid solution for 30 min, and then placed on the surface of a MH agar plate previously seeded with a 100 μl bacterial suspension containing 10^6^ CFU/ml test bacteria. The inhibitory zone was measured after 24 h incubation at 35°C.

### 2.7. Bacterial membrane damage

A LIVE/DEAD BacLight kit containing SYTO-9 and propidium iodide dyes purchased from Molecular Probes (Invitrogen, Carlsbad, CA, USA) was used to measure bacterial membrane damage. Briefly, bacteria were grown in MH broth to an OD_600_ of 0.3, which was equal to 10^7^ CFU/ml determined by plates count, and followed by asiatic acid treatments at 0.5, 1 or 2X MIC for 2 h at 37°C. After centrifugation at 10, 000 × g for 15 min, the pellet was collected and resuspended in a buffer containing 5 μM SYTO-9 and 30 μM propidium iodide in the dark for 15 min at room temperature. Green fluorescence, which reflected intact cell membranes, was read at 530 nm; and red fluorescence, which reflected damaged membranes, was read at 645 nm with an excitation wavelength at 485 nm. The ratio of green to red fluorescence intensities determined by a fluorescence microplate reader (Model MTP-601Lab, Hitachi High Technologies, Tokyo, Japan) was normalized to the bacterial sample without asiatic acid treatment.

### 2.8. Intracellular K^+^ concentration

The K^+^ concentration (ppm) released from the test bacteria was measured by a flame atomic absorption spectrometry (Model 5000, Perkin Elmer Inc., Norwark, CT, USA), according to the method of Li *et al*. [22]. Briefly, bacteria were grown in an MH broth to an OD_600_ of 0.3 (10^7^ CFU/ml). One mL of bacterial suspension was treated with asiatic acid at 0, 0.5, 1 or 2X MIC. After 2 h incubation at 37°C, and centrifugation at 10, 000 × g for 15 min, supernatants were collected, and the K^+^ concentration was measured.

### 2.9. Nucleotide leakage

The release of nucleotides was determined by a spectrophotometer (Model U-2000, Hitachi High Technologies, Tokyo, Japan) according to the method of Lou *et al*. [23]. Briefly, bacteria were grown in an MH broth to an OD_600_ of 0.3 (10^7^ CFU/ml). Asiatic acid at 0.5, 1 or 2X MIC was added into a 1 ml bacterial suspension, and followed by incubating for 2 h at 37°C. After centrifugation at 10,000 × g for 10 min, supernatants were collected, and the absorbance at 260 nm was measured.

### 2.10. Ground beef preparation

Beef semimembranosus muscle (top round) purchased locally was trimmed of all visible extramuscular fat. The beef muscle was then ground *via* a 4.5 m/m head on a grinder (Model TS-285, Ta-sin Ltd., Taichung City, Taiwan), and divided into several portions for the following experiments.

### 2.11. Antibacterial assay in ground beef

Asiatic acid at 0, 2, 4 or 8 mg was mixed with 100 g ground beef. One ml of each test bacterial culture at 10^4^ CFU/ml was added into the 100 g ground beef previously treated with asiatic acid. The inoculated ground beef was then mixed at low speed in a meat mixer (Model TS-383, Ta-sin Ltd., Taichung City, Taiwan) to assure uniform distribution of inoculum. Uninoculated beef samples were also used as negative controls. After 3 days storage at 25°C, 20 g of ground beef was homogenized with 100 ml of deionized water in a Waring blender (Model 31BL91, Sunwei Ltd., Taichung City, Taiwan) at high speed. Then, 1 ml beef homogenate was serially diluted with 9 ml of 0.5% peptone water, and a 0.1 ml portion of each dilution was spread on selective agar plates for enumeration. Plates were incubated at 35°C for 24 h, and colonies were counted and reported as a log of CFU/g ground beef.

### 2.12. Statistical analysis

All data were expressed as mean ± SD (n = 16). Differences among means were determined by the Least Significance Difference Test with significance defined at *P* < 0.05.

## 3. Results

**Fig. 2:**
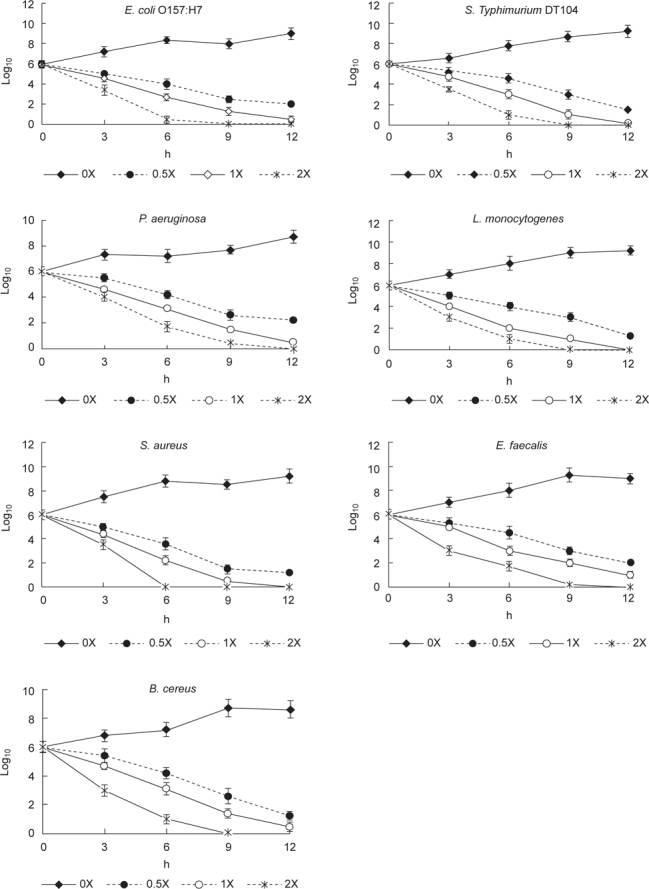
Time-kill curves of asiatic acid in broth. Asiatic acid at 0, 0.5, 1 or 2X MIC was added into a Mueller Hinton broth containing 10^6^ CFU/ml bacteria, followed by incubating at 35°C. The level of bacteria was measured at 0, 3, 6, 9 and 12 h. Data are expressed as mean ± SD (n = 16).

The MICs and MBCs of asiatic acid against the test bacteria are presented in Table 1. MICs were in the range of 20-40 μg/ml; MBCs were in the range of 32-52 μg/ml. The time-kill curves of asiatic acid at 0.5, 1 or 2X MICs against test bacteria strains are presented in Figure 2. After 6 h incubation, the bactericidal effects of asiatic acid upon test strains increased with increasing asiatic acid concentrations from 0.5 to 2 X MIC (*P* < 0.05). Asiatic acid at 2X MIC effectively reduced bacteria numbers from 6 log_10_ to < 2 log_10_ in all test bacteria within 6 h (*P* < 0.05). The influence of heat treatments upon the antibacterial effect of asiatic acid, determined by the inhibitory zone, is shown in Table 2. Compared with the 25°C asiatic acid treatment, the 50, 75, or 100°C heat treatments did not significantly affect the antibacterial activity of asiatic acid (*P* > 0.05).

**Fig. 3:**
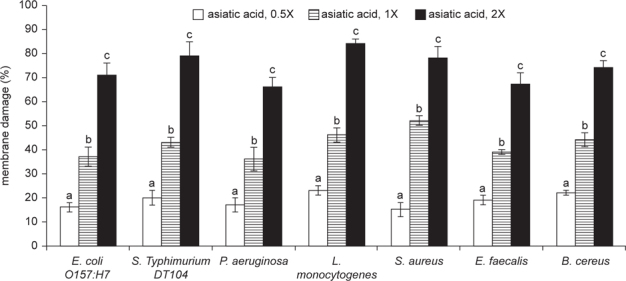
Damage caused by asiatic acid on bacterial membrane. Asiatic acid at 0.5, 1 or 2X MIC was added into a Mueller Hinton broth containing 10^7^ CFU/ml bacteria, and incubated for 2 h at 37°C. A *Bac*Light kit was used to determine membrane damage. Data are expressed as mean ± SD (n = 16). ^a-c^Means among bars without a common letter differ, *P* < 0.05.

**Fig. 4:**
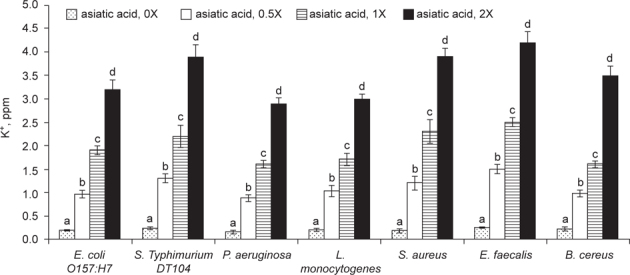
Effects of asiatic acid on bacterial intracellular K^+^ concentration (ppm). Asiatic acid at 0, 0.5, 1 or 2X MIC was added into a Mueller Hinton broth containing 10^7^ CFU/ml bacteria, and incubated for 2 h at 37°C. K^+^ concentration (ppm) was analyzed by a flame atomic absorption spectrometry. Data are expressed as mean ± SD (n = 16). ^a-d^Means among bars without a common letter differ, *P* < 0.05.

**Fig. 5:**
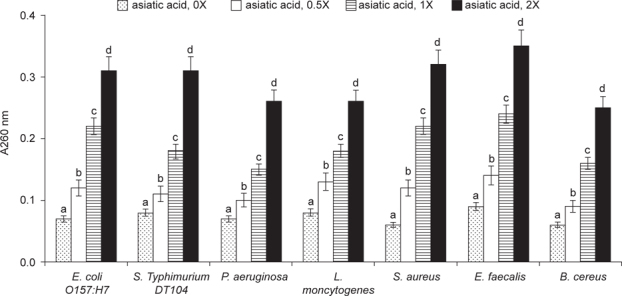
Effects of asiatic acid on bacterial nucleotide leakage. Asiatic acid at 0, 0.5, 1 or 2X MIC was added into a Mueller Hinton broth containing 10^7^ CFU/ml bacteria, and incubated for 2 h at 37°C. The released nucleotide level was determined by measuring absorbance at 260 nm. Data are expressed as mean ± SD (n = 16). ^a-d^Means among bars without a common letter differ, *P* < 0.05.

As shown in Figure 3, asiatic acid dose-dependently impaired membrane integrity (*P* < 0.05). Asiatic acid at 1 or 2X MICs led to 40-56% and 71-89% membrane damage, respectively, in test bacteria. Asiatic acid treatments also dose-dependently increased bacterial intracellular K^+^ concentrations in test bacteria (Figure 4, *P* < 0.05). Asiatic acid at 1 or 2X MICs led to 1.5-2.4 ppm and 2.9-4.1 ppm K+ release within 2 h, respectively. As shown in Figure 5, asiatic acid treatments caused bacterial nucleotide leakage (*P* < 0.05). Asiatic acid at 1X MIC increased 1-2.2 folds nucleotide leakage; and at 2X MIC increased 2.7-4.3 folds nucleotide leakage in test bacteria within 2 h.

The antibacterial effects of asiatic acid in ground beef are presented in Table 3. After 3 days of storage at 25°C, the addition of asiatic acid dose-dependently inhibited the growth of test bacteria in ground beef (*P* < 0.05), in which 8 mg of asiatic acid treatments led to bacterial levels in ground beef lower than 2 log_10_.

## 4. Discussion

The antibacterial activity of asiatic acid against *E. coli, B. subtilis* and *S. sonnei* has been reported before [13]. The results of our present study extend the inhibitory effects of this agent toward other Gram-negative and Gram-positive foodborne bacterial pathogens including *S. Typhimurium* DT104, *P. aeruginosa, L. monocytogenes, S. aureus, E. faecalis*, and *B. cereus*. The MICs of this agent against those bacteria were ≤ 40 μg/ml. Furthermore, we used ground beef as a food model to evaluate the antibacterial potency of asiatic acid. The results reveal that the addition of asiatic acid markedly inhibits bacterial growth in ground beef. These findings indicate that asiatic acid is an effective wide spectrum antibacterial agent against seven foodborne bacterial pathogens in media and ground beef. Thus, this agent could be considered as a potent additive in foods to prevent bacterial contamination. In addition, we found that heat treatments of up to 100°C did not affect the inhibitory effects of asiatic acid against test bacteria. This heat-resistant property benefits its application for foods requiring a heating process of 100°C or lower. That is, this compound is applicable for raw and cold foods, as well as food products treated with high temperature, short time pasteurization.

**Table 2:** Inhibitory zone (mm) of asiatic acid against seven bacteria at 25, 50, 75 and 100°C. The inhibitory zone was determined by disc diffusion method.

Bacteria	25°C	50°C	75°C	100°C
*E. coli* O157:H7	33 ± 3^a^	31 ± 5^a^	32 ± 3^a^	30 ± 4^a^
*S*. Typhimurium DT104	25 ± 3^a^	23 ± 5^a^	26 ± 2^a^	25 ± 4^a^
*P. aeruginosa*	24 ± 5^a^	27 ± 4^a^	25 ± 3^a^	23 ± 2^a^
*L. monocytogenes*	28 ± 2^a^	26 ± 4^a^	30 ± 4^a^	27 ± 5^a^
*S. aureus*	30 ± 4^a^	33 ± 3^a^	29 ± 5^a^	32 ± 2^a^
*E. faecalis*	35 ± 2^a^	36 ± 3^a^	38 ± 4^a^	35 ± 5^a^
*B. cereus*	22 ± 2^a^	20 ± 3^a^	23 ± 4^a^	21 ± 4^a^

Data are expressed as mean ± SD (n = 16).

^a^Means in a row without a common letter differ, *P* < 0.05.

**Table 3:** Bacterial level (log CFU/g) in 100 g of ground beef treated with asiatic acid at 0, 2, 4 or 8 mg after 3 days of storage at 25°C.

Bacteria	Log CFU/g			
	asiatic acid, 0	asiatic acid, 2	asiatic acid, 4	asiatic acid, 8
*E. coli* O157:H7	7.4 ± 0.4^d^	4.7 ± 0.2^c^	1.6 ± 0.4^b^	0.5 ± 0.3^a^
*S*. Typhimurium DT104	8.1 ± 0.5^d^	5.2 ± 0.6^c^	2.3 ± 0.5^b^	0.7 ± 0.1^a^
*P. aeruginosa*	7.4 ± 0.6^d^	4.4 ± 0.5^c^	1.8 ± 0.4^b^	0.4 ± 0.2^a^
*L. monocytogenes*	7.7 ± 0.5^d^	4.5 ± 0.2^c^	1.9 ± 0.3^b^	0.3 ± 0.1^a^
*S. aureus*	8.0 ± 0.7^d^	5.3 ± 0.5^c^	2.4 ± 0.2^b^	0.4 ± 0.2^a^
*E. faecalis*	7.5 ± 0.3^d^	4.4 ± 0.4^c^	1.7 ± 0.6^b^	0.3 ± 0.2^a^
*B. cereus*	7.8 ± 0.6^d^	4.5 ± 0.3^c^	2.0 ± 0.4^b^	0.6 ± 0.3^a^

Data are expressed as mean ± SD (n = 16).

^a-d^Means in a row without a common letter differ, *P* < 0.05.

Bacterial membrane integrity is important not only for bacteria’s self-protection but also for the functions of membrane-associated enzymes responsible for energy generation, respiration, and redox balance [24, 25]. Thus, bacterial membrane rupture easily impairs these critical functions, which in turn affects the bacteria’s survival and growth. In our present study, asiatic acid destroyed the membrane integrity of test bacteria, which subsequently interfered with these above functions, and caused bacterial apoptosis. Potassium ions are the most abundant cations in bacteria such as *E. coli*, and their homeostasis is regulated by K^+^ transporters such as Kdp [26]. These cations are involved in many aspects of bacterial physiological actions including growth, survival, and virulence [27]. Thus, the loss of K^+^ as we observed was definitely detrimental upon the bacteria’s growth and survival. We found that asiatic acid treatments increased K^+^ release from cytoplasm and/or mitochondria in test bacteria. These results indicate that asiatic acid induces irreversible damage of the cytoplasmic membranes, and disturbed K^+^ homeostasis in test bacteria. It is well known that nucleotides such as DNA and RNA are crucial factors responsible for cell repair and replication [28]. The release of nucleotides and their derived compounds including DNA and RNA from bacteria can be quantified by monitoring their absorbance at 260 nm because these substances possess strong UV absorption at this wavelength [29, 30]. In our present study, asiatic acid treatments effectively promoted nucleotides release from the intracellular compartments of test bacteria, which was reflected in their increased absorbance at 260 nm. Since nucleotides were released, the observed death in asiatic acid treated bacteria can be explained. These findings imply that asiatic acid is able penetrate into bacteria, cause damage in DNA-containing organelles like mitochondria or nuclei, which in turn alters nuclear stability. Our above findings indicate that asiatic acid exerts its antibacterial actions through causing membrane damage, and increasing K^+^ and nucleotides release in test bacteria.

Asiatic acid is a triterpenoid naturally occurring in many edible plant foods [31]. Based on its natural, tasteless, and odorless properties, this agent might be safe and not affect food flavor. In our present study, 8 mg of asiatic acid in 100 g ground beef was equal to 8 ppm, and exhibited markedly anti-bacterial effects. This dosage is not considered high. Thus, the application of asiatic acid to prevent bacterial contamination in foods seems feasible. Our results support the idea that this agent could be applied to foods against bacterial contamination; or used as a bactericide in farms and/or slaughter houses to enhance environmental sanitation. However, further study regarding its safety and possible side effects is necessary before it is used for food preservation. In addition, several animal studies have reported that the dietary intake of asiatic acid could provide anti-diabetic, anti-hyperlipidemic, and hepatic protection *via* its anti-oxidative activity [32]. It is highly possible that using this triterpene as an antioxidant also improves foods’ oxidative stability and benefits food preservation.

In conclusion, asiatic acid dose-dependently inhibited the growth of *E. coli* O157:H7, *S*. Typhimurium DT104, *P. aeruginosa, L. monocytogenes, S. aureus, E. faecalis*, and *B. cereus* in medium and in ground beef. Asiatic acid could impair membrane integrity, increase the release of potassium ions and nucleotides in test bacteria. These findings support the contention that asiatic acid is a potent agent to prevent foods from being contaminated by the aforementioned bacteria.

## Conflicts of interest statement

The authors declare that there are no conflicts of interest.

## Abbreviation

CFU: colony forming unit
DMSO: dimethyl sulphoxide
MBC: minimum bactericidal concentration
MH: Mueller Hinton
MIC: minimal inhibitory concentration
OD: optical density
